# Urease and nitrification inhibitors with pig slurry effects on ammonia and nitrous oxide emissions, nitrate leaching, and nitrogen use efficiency in perennial ryegrass sward

**DOI:** 10.5713/ab.21.0046

**Published:** 2021-04-23

**Authors:** Sang Hyun Park, Bok Rye Lee, Tae Hwan Kim

**Affiliations:** 1 Department of Animal Science, College of Agriculture & Life Science, Chonnam National University, Gwangju 61186, Korea; 2 Biotechnology Research Institute, Chonnam National University, Gwangju 61186, Korea

**Keywords:** *Lolium perenne*, Nitrification Inhibitor, Pig Slurry, Regrowth, Urease Inhibitor

## Abstract

**Objective:**

The present study was conducted to assess the effect of urease inhibitor (hydroquinone [HQ]) and nitrification inhibitor (dicyandiamide [DCD]) on nitrogen (N) use efficiency of pig slurry for perennial ryegrass regrowth yield and its environmental impacts.

**Methods:**

A micro-plot experiment was conducted using pig slurry-urea ^15^N treated with HQ and/or DCD and applied at a rate of 200 kg N/ha. The flows of N derived from the pig slurry urea to herbage regrowth and soils as well as soil N mineralization were estimated by tracing pig slurry-urea ^15^N, and the N losses via ammonia (NH_3_), nitrous oxide (N_2_O) emission, and nitrate (NO_3_^−^) leaching were quantified for a 56 d regrowth of perennial ryegrass (*Lolium perenne*) sward.

**Results:**

Herbage dry matter at the final regrowth at 56 d was significantly higher in the HQ and/or DCD applied plots, with a 24.5% to 42.2% increase in ^15^N recovery by herbage compared with the control. Significant increases in soil ^15^N recovery were also observed in the plots applied with the inhibitors, accompanied by the increased N content converted to soil inorganic N (NH_4_^+^+NO_3_^−^) (17.3% to 28.8% higher than that of the control). The estimated loss, which was not accounted for in the herbage-soil system, was lower in the plots applied with the inhibitors (25.6% on average) than that of control (38.0%). Positive effects of urease and/or nitrification inhibitors on reducing N losses to the environment were observed at the final regrowth (56 d), at which cumulative NH_3_ emission was reduced by 26.8% (on average 3 inhibitor treatments), N_2_O emission by 50.2% and NO_3_^−^ leaching by 10.6% compared to those of the control.

**Conclusion:**

The proper application of urease and nitrification inhibitors would be an efficient strategy to improve the N use efficiency of pig slurry while mitigating hazardous environmental impacts.

## INTRODUCTION

Nitrogen (N) is an essential nutrient as a key limiting factor of the growth and development of plants in agricultural ecosystems [1]. Incremental increases in global crop yields during the past several decades has mainly been dependent on the increasing application of synthetic N fertilizers. Animal manures have long been used as alternative organic N fertilizers. Most of the N in feces is present in organic form, while in urine, 65% to 90% of the N is present as urea [2]. In Korea, pig slurry is the most viable recycling option and represents more than 80% of all recycled animal manure [3] because pig farms usually have little or no arable land for forage production.

The amount of N supplied to agro-ecosystems is often higher than N uptake by crops. An excessive N input leads to N losses via volatilization of ammonia (NH_3_), emission of nitrous oxide (N_2_O), and leaching of NO_3_^−^-N, which pose a significant threat to the environmental quality of the atmosphere and aquatic systems [4]. Thus, management of N nutrition is important to increase crop productivity and control environmental pollution. The chemical or organic N applied to the soil, mainly in form of urea, hydrolyze into NH_4_^+^, hydroxyl, and carbonate ions by the microbial urease mediation. The NH_4_^+^ produced then converts to NH_3_, which can be lost through volatilization under alkaline conditions. If soil condition does not favor volatilization, NH_4_^+^ can either be held in the soil via cation exchange or converted to NO_3_^−^, leading to N losses through leaching or denitrification. A proportion of volatilized and deposited NH_3_ can generate N_2_O, which is a long-lasting greenhouse gas, through both nitrification, in which aerobic oxidation of NH_4_^+^ to NO_2_^−^ and further NO_3_^−^, and denitrification, in which NO_3_^−^ is reduced to N_2_O [5].

Various management practices and technologies have attempted to enhance N fertilizer use efficiency while minimizing N losses to the environment. One of the strategies is the use of inhibitors of urea hydrolysis (urease inhibitors) and of ammonia oxidation (nitrification inhibitors), which have been shown to be effective in enhancing N use efficiency by delaying nitrification/denitrification [6,7]. The efficacy of urease and/or nitrification inhibitors in mitigating NH_3_ and N_2_O emissions varies with soil pH [8], type and level of applied N sources [9], the concentration of inhibitors [10], soil texture [11], as well as climatic factors such as rainfall [12]. Martins et al [7] showed that the urease and nitrification inhibitors enhanced urea-^15^N recovery by maize and increased grain yield. In a meta-analysis with 111 datasets from 39 studies [5], nitrification inhibitors are effective in reducing N_2_O emissions with the highest inhibitory effect in grassland and followed by cropland, upland, and paddy. Li et al [13] reported that N_2_O emission was efficiently lower in urea together with urease and nitrification inhibitors than with either a single urease or nitrification inhibitor. However, the flux of N derived from animal manure to pasture plants and soil has not been fully elucidated. In addition, few studies have assessed the effects of inhibitors on gaseous emissions and nitrate leaching from animal manure-based N [14].

In the present study, we hypothesized that the synergistic effect of urease inhibitor (hydroquinone [HQ]) and nitrification inhibitor (dicyandiamide [DCD]) may improve N use efficiency of pig slurry and minimize the N losses to the environment by regulating N mineralization processes in soil. To test this hypothesis, the turnover of pig slurry-urea ^15^N and its flow into the plant and soil inorganic N components were directly quantified while accounting for N losses to the environment (NH_3_ and N_2_O emissions and nitrate leaching). The resulting data were interpreted regarding the effectiveness of HQ and/or DCD.

## MATERIALS AND METHODS

### Experimental design

The study was based on field experiments conducted on a permanent grass sward consisting mainly of perennial ryegrass (*Lolium perenne*) on sandy loamy soil. The soil chemical properties of the experimental site are presented in Table 1. During the experimental period, the typical climate was temperate with high humidity, with an average temperature of 22.5°C and total precipitation of 420 mm. Four treatments of slurry application were compared: i) only pig slurry as a control, ii) HQ treatment (pig slurry + urease inhibitor [HQ, C_6_H_6_O_2_]), iii) DCD treatment (pig slurry + nitrification inhibitor [DCD, NH_4_F]), and iv) HQ and DCD combination treatment. The experiment in a randomized complete block design consisted of four replications. Each treatment plot measured 2.5 m×10 length experiment and contained 12 micro-plots (0.5 m×0.5 m) for monitoring the fate of ^15^N-labeled pig slurry. To prevent surface runoff and contamination by slurry application, there was a 2 m margin between plots with a 0.45 m metal retainer inserted 30 cm deep soil. The bottomless acrylic chambers (0.2 m diameter and 0.3 m length) were used for collecting gas samples and suction cups (P80, eco Tech, Bonn, Germany) for collecting leachate samples.

### Pig slurry treatments and ^15^N labeling

The pig slurry was obtained from pig livestock farm and stored in concrete tanks at ambient temperature for approximately 1 week. Four different 400 L plastic containers filled with pig slurry were mixed with 0.3% HQ or 5% DCD of the total-N in pig slurry, respectively. The slurry urea fraction of four treatments were labeled by thoroughly mixing with highly enriched ^15^N urea (98% ^15^N atom excess) just before application. The applied pig slurries were analyzed at the time of application. The pig slurry contained on average (kg/m^3^) 1.58±0.32 total N, 0.98±0.12 urea, 0.199±0.02 NH_4_^+^-N and 0.143±0.01 NO_3_^−^-N with ^15^N-urea enrichment of 5.001±0.012 atom %. Treated pig slurry at a rate of 200 kg N/ha (e.g., 316 L per 25 m^2^ plot, which contained 95.8 kg P/ha and 127 kg K/ha) was applied after herbage was cut at 50 mm above ground level [3].

### Herbage, soil, gases, and leachate sampling

The herbage sample was harvested from four randomly placed micro-plots by cutting manually, and the remained stubble was approximately 50 mm. About 500 g of collected herbage sample was chopped into 20 mm long segments, and then lyophilized, ground, and stored in a vacuum desiccator for chemical analysis. The soil samples were collected by soil cores (0 to 0.3 m depth) randomly using a 0.3 m diameter tube auger in the same micro-plots that herbage sampling place. The collected soil samples were air-dried, ground, and sieved to <0.15 mm. The herbage and soil sampling were done at 7, 14, and 56 d after pig slurry application, respectively.

Airtight acrylic chambers were located to 50 mm depth soil in each experimental plot for gas sampling. To collect NH_3_ emission, the acid trap system method was used as described by Ndegwa et al [15] with modifications. Each chamber was connected to NH_3_-N trapping bottles containing 150 mL of 0.2 mol/L H_2_SO_4_ and a vacuum system to pull air through the chambers. The NH_3_-N traps a constant rate of 1.5 L per minute. Each chamber was closed with silicon sealing and clamped for 24 hours. The NH_3_ sampling in each treatment block was done at the same time over 1 hour to avoid the impact of extraneous gases. The N_2_O gas was collected by using a syringe before NH_3_ emission sampling and then stored in 10 mL of vacutainer tuber. The gas of NH_3_ and N_2_O was collected daily for the first 14 d, then at intervals 1 to 2 weeks. The leachate samples were obtained by suction cups in each plot at a depth of 0.5 m for NO_3_^−^-N analysis. Soil water samples were obtained by applying a tension −250 hPa. A sampling of NO_3_^−^-N was done weekly and stored at −20°C.

### Measurements and chemical analysis

The herbage was harvested from each micro-plot and converted to kg/ha. To calculate the N recovery in herbage (kg N/ha), the converted estimate was multiplied by the N concentration determined in the subsamples. The stable isotope ratio mass spectrometer (IRMS, IsoPrime, GV Instrument, Manchester, UK) was used for measuring the total N content and ^15^N atom % of herbage, soil, and pig slurry samples. Inorganic nitrogen was extracted with 2 M KCl and the NH_4_^+^-N was determined by distillation in an alkaline medium (MgO). The same procedure was used for NO_3_^−^-N after reduction with Devarda’s alloy. The N liberated from each distillation was collected in H_2_SO_4_ and then evaporated to dryness to analyze the determination of ^15^N atom % excess of each N fraction. The total N and inorganic N (NH_4_^+^-N and NO_3_^−^-N) concentration in soil samples were converted to kg N/ha using soil bulk density. To determination of NH_3_ volatilization, the solution collected by acid traps in the form of (NH_4_)_2_SO_4_ was quantified by a colorimetric determination with ammonium color reagent (Nessler’s reagent, Sigma, 72190; St. Louis, MO, USA) as described by Kim and Kim [16]. N_2_O concentration in gas samples collected was determined using a gas chromatograph (GC-7890A, Agilent Technologies, Santa Clara, CA, USA) equipped with a thermal conductivity detector (TCD) and with a HP-Plot 5A column (30 m×0.53 mm×25 μm) under the following conditions: column oven temperature 40°C; injector temperature 100°C; detector temperature 300°C; carrier gas helium (2 mL/min). The N_2_O fluxes were calculated as described by Guo et al [6]. The concentration of NO_3_^−^-N leaching was determined by ion chromatography DX 120 Dionex as described by Hamonts et al [17]. The total NH_3_, N_2_O emission, and NO_3_^−^ leaching over the entire experimental period were calculated by the sum of daily measurements.

The determined ^15^N atom % excess abundances in the total N and inorganic N fractions in herbage and soil samples were converted to relative specific activity and the amount of N derived from pig slurry urea (NdfSU) in herbage samples was calculated as described by Park et al [18]. The ratio between the NdfSU and the quantity of applied N was applied for percentage of slurry urea-N recovery in the total N, NH_4_^+^, and NO_3_^−^ fractions in herbage and soil. Therefore, the portion not recovered in herbage and soil indicate the percentage of loss.

### Statistical analysis

Analysis of variance was conducted to assess the effects of urease and/or nitrification inhibitors with pig slurry at each sampling time on herbage yield, N uptake, gas emissions, leaching, and the fate of slurry urea-N. Statistical analysis were conducted using the SAS 9.1.3 software.

## RESULTS

### Dry matter, total N and N amount derived from slurry urea in herbage

**Figure 1:**
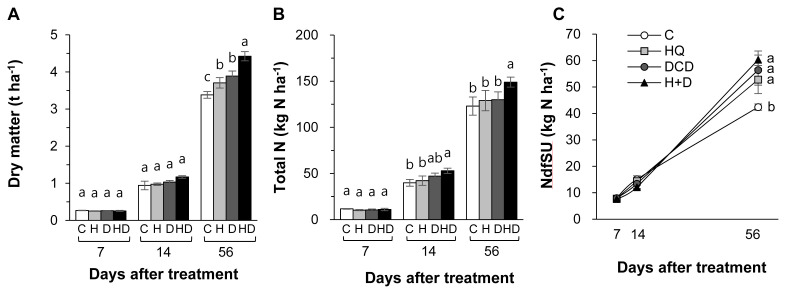
Dry matter (A), total nitrogen (B) and the amount of N derived from pig slurry urea (NdfSU) (C) in herbage as affected by pig slurry with urease inhibitor (HQ, H) and/or nitrification inhibitor (DCD, D) during regrowth of perennial ryegrass sward. HQ, hydroquinone; DCD, dicyandiamide. ^a–c^ Different letters indicate significant differences at p<0.05 according to the Duncan’s multiple range test.

Herbage dry matter (DM) was not influenced by the application of urease and nitrification inhibitors during the first 14 d. However, at final regrowth at 56 d, combined application of HQ and DCD (HQ+DCD) induced the highest herbage DM yield (+30.8%), followed by DCD (+14.5%) and HQ (+9.6%) single applications, compared to that in the control (only pig slurry applied) (Figure 1A). Total N content in herbage increased only in the HQ+DCD plot from 14 d, in which it was 21% to 33% higher than that in the control (Figure 1B). The amount of NdfSU in herbage at the final regrowth at 56 d significantly increased only at 56 d by 24.5%, 33.0%, and 42.2% in the HQ, DCD, and HQ+DCD applied plots, respectively, compared to that of the control (p<0.001) (Figure 1C). However, among the HQ, DCD, and HQ+DCD applied plots, there were no significant differences.

### Soil N dynamics

**Figure 2:**
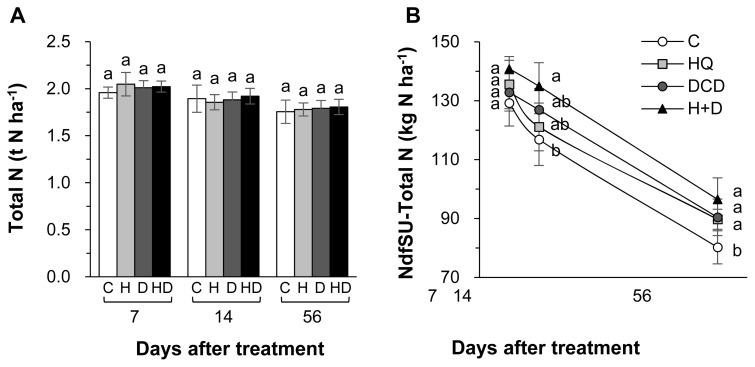
Total N (A) and the amount of N derived from pig slurry urea (NdfSU) (B) in soil as affected by pig slurry with urease inhibitor (HQ, H) and/or nitrification inhibitor (DCD, D) during regrowth of perennial ryegrass sward. HQ, hydroquinone; DCD, dicyandiamide. ^a,b^ Different letters indicate significant differences at p<0.05 according to the Duncan’s multiple range test.

**Figure 3:**
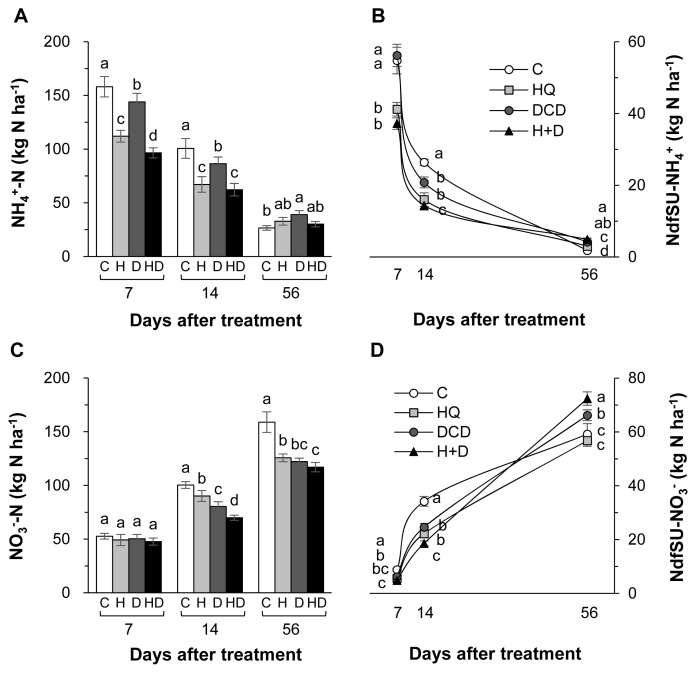
Ammonium-N (NH_4_^+^-N) (A), nitrate-N (NO_3_^−^-N) (C) and the amount of N derived from pig slurry urea (NdfSU-NH_4_^+^, B) and NdfSU-NO_3_^−^ (D) in soil as affected by pig slurry with urease inhibitor (HQ, H) and/or nitrification inhibitor (DCD, D) during regrowth of perennial ryegrass sward. HQ, hydroquinone; DCD, dicyandiamide. ^a–d^ Different letters indicate significant differences at p<0.05 according to the Duncan’s multiple range test.

The inhibitors (HQ and/or DCD) did not affect the total N pool size in soil throughout the regrowth period (Figure 2A). However, the NdfSU in the soil at 56 d increased by 11.8%, 12.7%, and 20.3%, respectively, in the HQ, DCD, and HQ+ DCD plots compared with the control (Figure 2B). The content of NH4+-N in soil was significantly reduced by the application of the inhibitors during the first 14 d with a stronger effect of HQ, whereas it was higher than control in the DCD plot or recovered to the control level in the HQ and HQ+DCD plot at 56 d (Figure 3A). The amount of N derived from slurry urea in the soil NH_4_^+^ fraction (NdfSU-NH_4_^+^) during the first 14 d of regrowth showed a similar pattern, with a significant reduction following HQ and/or DCD application (Figure 3B). The final NdfSU-NH_4_^+^ at 56 d was the highest in the HQ+DCD plot (4.9 kg N/ha) and followed by the DCD (4.2 kg N/ha), HQ (3.0 kg N/ha), and control (1.8 kg N/ha) plot. The content of NO_3_^−^-N in the soil was lower in all plots applied with the inhibitors than that in the control throughout whole the regrowth period (Figure 3C). The amount of N derived from slurry urea in the soil NO_3_^−^ fraction (NdfSU-NO_3_^−^) also remained lower until 14 d. The final NdfSU-NO_3_^−^ at 56 d significantly increased in the DCD (+14.5% compared to that of the control) and HQ+ DCD (+22.5%) plots (Figure 3D).

### Recovery of pig slurry-urea ^15^N

The percentage of pig slurry-urea ^15^N recovered in herbage averaged over all treatments gradually increased from 3.9% (at 7 d) to 26.5% (at 56 d), whereas the soil ^15^N recovery decreased from 67.3% to 44.8% over the same period (Table 2). Thus, at the end of regrowth (56 d after pig slurry application), the herbage ^15^N recovered was higher in the HQ and/or DCD plots than in the control plots, with no significant difference among the three inhibitors treatments. The soil ^15^N recovery was also significantly increased by the inhibitor treatments. The percentage of pig slurry-urea ^15^N recovered in the soil NH_4_^+^ and NO_3_^−^ fractions were also increased by application of urease and nitrification inhibitors, with the combined application of HQ and DCD showing a stronger effect. The percentage of ^15^N recovered in the soil inorganic N (NH_4_^+^ and NO_3_^−^) pool at 56 d was 22.4%, 33.6%, 31.5%, and 36.2% in the control, HQ, DCD, and HQ+DCD plots, respectively. The estimated N loss at the final regrowth was in the range of 21.6% to 28.4% in the plots applied with the inhibitors, and it was relatively higher in the control treatment (38.0%).

### NH**_3_**, N**_2_**O emission, and NO**_3_**^−^ leaching

**Figure 4:**
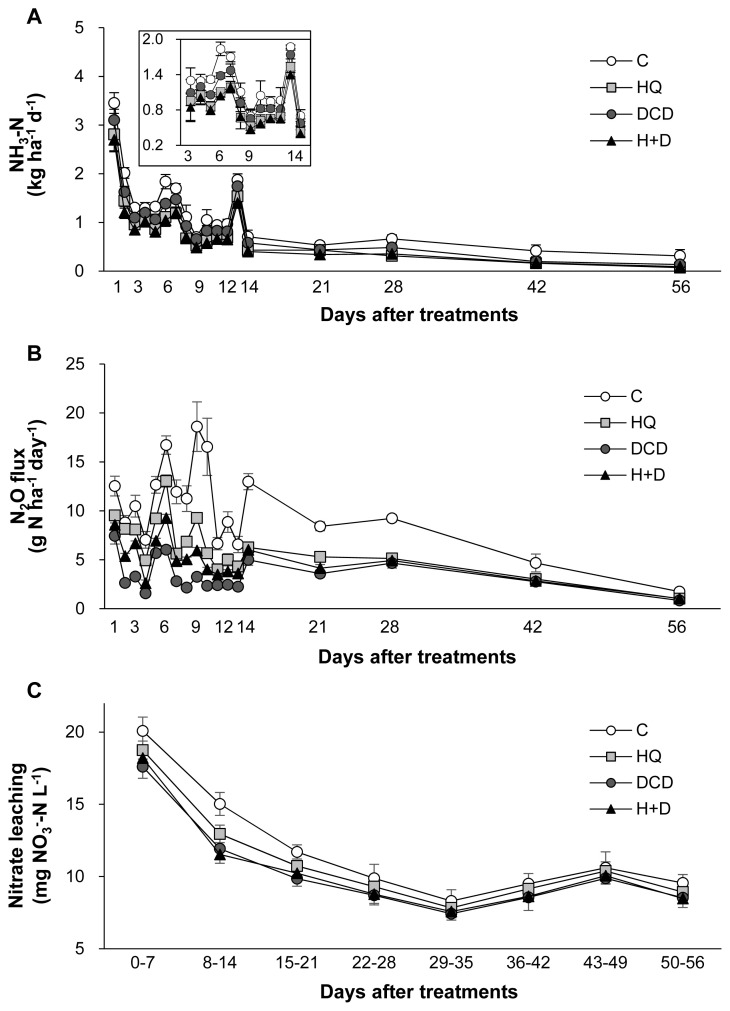
Daily emissions of ammonia (A) and nitrous oxide (B) and weekly accumulative nitrate concentration in leachate (C) from the control (○), urease inhibitor (HQ, ■), nitrification inhibitor (DCD, ●) and the combination of HQ and DCD (H+D, ▲) during regrowth of perennial ryegrass sward. HQ, hydroquinone; DCD, dicyandiamide.

**Figure 5:**
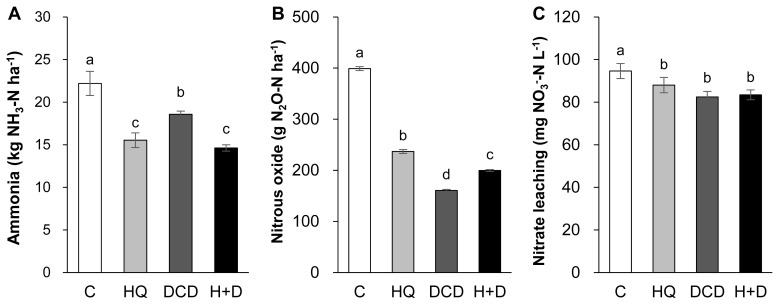
Total emission of ammonia (A) and nitrous oxide (B) and nitrate leaching (C) as estimated by cumulative amount for 56 days of regrowth. ^a–d^ Different letters indicate significant differences at p<0.05 according to the Duncan’s multiple range test.

The N losses through gaseous emissions of ammonia (NH_3_) and N_2_O as well as aqueous nitrate (NO_3_^−^) leaching were quantified. On average, 58.8% of total NH_3_ emission during a 56 d period of regrowth occurred within the first 14 d after application of pig slurry to the soil. The daily NH_3_ emission during this period was relatively lower in the HQ and DCD +HQ plots than in the control and DCD plots (Figure 4A). Cumulative NH_3_ emission during 56 d of regrowth decreased by 30.0%, 16.3%, and 34.1% in the HQ, DCD, and DCD+HQ plots compared with the control plots (Figure 5A). Consistent with NH_3_ emission, significant effects of inhibitors in reducing daily N_2_O emission was observed, with a stronger effect observed for DCD (Figure 4B). N_2_O emission in all treatments decreased to near the background level after 56 d of application of pig slurry. Cumulative N_2_O emission throughout the experimental period decreased by 40.7%, 59.8%, and 50.0% in the HQ, DCD, and DCD+HQ plots compared with the control plot (Figure 5B). The weekly cumulative NO_3_^−^ leaching was lower in the plots applied with the inhibitors, especially prior to 21 d after pig slurry application. Overall DCD application (e.g., DCD and HQ+DCD treatment) was more effective in reducing NO_3_^−^ leaching (Figure 4C). Cumulative NO_3_^−^ leaching for the whole experimental period declined by 7.0%, 12.9%, and 11.8% in the HQ, DCD, and DCD+HQ plots, respectively, compared with the control plots (Figure 5C).

## DISCUSSION

### Regrowth and pig slurry-urea ^15^N recovery in herbage

The efficacy of different types of urease inhibitors [HQ, phenyl phosphorodiamidate (PPDA), and N-(n-butyl) thiophosphoric triamide (NBPT)] and nitrification inhibitors (DCD, 3, 4-dimethylpyrazole phosphate [DMPP], Nitrapyrin, and thiosulphate) have been tested to improve N use efficiency while minimizing N losses to the environment. For instance, a meta-analysis of 113 field experiments showed that the effectiveness of various urease and nitrification inhibitors was relatively consistent across land use types in both chemical and organic N fertilizers [19]. In this context, we focused on urease inhibitor HQ and nitrification inhibitor DCD because HQ is lower cost [20], DCD is less volatile, and easily blended with fertilizers [5]. In the present study, single or combined HQ and DCD treatments did not influence the amount of NdfSU in herbage during the first 14 d of regrowth, whereas at the final regrowth (56 d) positive effects of HQ and/or DCD were observed, at which NdfSU was enhanced by 33.2% (on average 3 inhibitor treatments) compared with the control (Figure 1C). Consistent with NdfSU, the final herbage DM at 56 d significantly increased in the HQ and/or DCD applied plots (Figure 1A). This indicated that inorganic N might be more available during the later period of regrowth due to delayed hydrolysis of urea in pig slurry by HQ, and reduced oxidation of NH_4_^+^ to NO_3_^−^ by DCD. In addition, these results indicated that early regrowth might be less dependent on exogenous N uptake by plants [21]. At final regrowth (56 d), the recovery of pig slurry-urea ^15^N varied within the range of 26.4% to 30.2% in the HQ and/or DCD applied plots, which was higher than that of the control (21.2%) (Table 2). By using ^15^N tracing, Choi et al [22] revealed that N is produced from organic amendments and N uptake was more pronounced during the later growth period of Chinese cabbage.

### Soil mineralization and pig slurry-urea ^15^N recovery

Plant uptake of N released from animal manure gradually increases with progressing regrowth of perennial grasses [3,18,23]. In the present study, at the final regrowth (56 d), we found a significant increase in herbage N content in the HQ+DCD plot, and NdfSU in herbage of all plots applied with the inhibitors (Figure 1B). However, the soil total N content was not affected by the inhibitors throughout the experimental period (Figure 2A). This indicates that enhanced N uptake and herbage growth in the HQ and/or DCD applied plots are due to inorganic N released from organic N rather than the N pool size in soil [3,23]. The NdfSU in the soil total N gradually decreased from 134.6 (at 7 d) to 89.2 kg N/ha (at 56 d) (based on average values of 4 treatments), corresponding to a decrease of ^15^N recovery in soil from 67.0% to 44.8% (Figure 2). This implies that N released from the applied urea in pig slurry dilutes the soil inorganic N pool, which is available for herbage regrowth. However, the NdfSU in herbage was not significantly affected by HQ and/or DCD application during the first 14 d of regrowth, although the amount of N derived from the pig slurry-urea in the soil NH_4_^+^ (NdfSU-NH_4_^+^) or NO_3_^−^ fractions (NdfSU-NO_3_^−^) decreased in the HQ and/or DCD treatments from 7 d (Figure 3). This may reflect a common N utilization pattern during the early regrowth characterized by low exogenous N uptake because shoot regrowth during this period depends on a large portion of endogenous N rather than exogenous N uptake [21]. In addition, during the first 7 d of regrowth, urea ^15^N in pig slurry was mineralized mainly to NH_4_^+^-N, which accounted for 63.6% to 88.6% of total NdfSU in the soil mineral N (sum of NdfSU-NH_4_^+^ and NdfSU-NO_3_^−^) (Figure 3). The NdfSU-NH_4_^+^ was lower in the plots applied with the inhibitors, especially in the presence of HQ (e.g., HQ and HQ+ DCD treatments) during the first 14 d, suggesting that HQ delayed the hydrolysis of urea in pig slurry [7]. The NdfSU-NH_4_^+^ in soil slowed down with progressing regrowth with an opposite increase in the NdfSU-NO_3_^−^ (Figure 3B, D), reflecting nitrification of the NH_4_^+^ released from pig slurry-urea. The NdfSU-NO_3_^−^ in the soil at 56 d of regrowth was significantly higher in the presence of the inhibitors, especially in the presence of DCD (e.g., DCD and HQ+DCD treatments), compared with the control (Figure 3). At the final regrowth (56 d), the N content converted to soil inorganic N from pig slurry-urea (NdfSU-NH_4_^+^ + NdfSU-NO_3_^−^) was higher in the presence of DCD (70.4 to 77.3 kg N/ha) compared to that of control (60.0 kg N/ha) (Figure 3). Retention of higher NdfSU-NH_4_^+^ and NdfSU-NO_3_^−^ in the soils amended the inhibitors may reflect the active onset of hydrolysis of urea and subsequent nitrification during the latter regrowth period when the uptake of exogenous N strongly occurs as a primary N source for the herbage regrowth [21]. Thus, enhanced final regrowth yield (Figure 1A) and higher NdfSU in herbage at 56 d (Figure 1C) in the HQ and/or DCD plots are certainly attributed to the higher availability of N released from pig slurry, as evidenced by higher percentages of urea ^15^N recovered in the soil inorganic N, i.e., 38.6%, 33.6%, and 31.5% of the ^15^N applied in the DCD, HQ, and HQ+DCD plots, respectively, compared with the control (22.4%). Many studies have shown positive effects of urease and/or nitrification inhibitors on plant nutrient availability in soil, enhancing yields of annual crops [24,25] and herbage in perennial grasslands [26].

### N losses via NH**_3_**, N**_2_**O emissions, and NO**_3_**^−^ leaching

Although the N in animal manure, especially for urine where urea makes up 65% to 90% of N, is economically attractive, it may also result in environmental pollution via N losses as odorous gases (e.g., NH_3_ and H_2_S), greenhouse gases (e.g., N_2_O and CH_4_) and NO_3_^−^-N leaching when inefficiently used by plants. The options using inhibitors of the N cycle, such as urease and nitrification inhibitors, have been evaluated to mitigate N losses from chemical N fertilizers, mainly urea [25,27] and from animal manure [28]. The present ^15^N recovery data has shown that 38.0%, 27.0%, 28.4%, and 21.6% of applied N were unaccounted in the control, HQ, DCD, and HQ+DCD plots, respectively (Table 2). In this study, these percentages were designated as the estimated N loss and the noxious N losses to NH_3_, N_2_O emission, and NO_3_^−^ leaching.

The application of animal manure causes NH_3_ volatilization via the N decomposition present in the feces and urea hydrolysis. Urea is hydrolyzed by urease and produces NH_3_ and carbonic acid. Thus, significant enhancement of daily NH_3_ emission after animal manure application has been observed in various cropping systems [23,29]. In the present study, daily NH_3_ emission significantly reduced in the presence of HQ (e.g., HQ and HQ+DCD plots) during the first 14 d (Figure 4A), when a large portion of NH_3_ emission (58.8%, averaged over 4 treatments, of total NH_3_ emission) occurred (Figure 5A). This result indicates that the urease inhibitor HQ efficiently abates the pool of NH_4_^+^ (Figure 3A) by slowing the hydrolysis of urea, which alleviates the subsequent NH_3_ emission, especially during the early period. Zhengping et al [20] estimated in the laboratory incubation that a urease inhibitor NBPT decreased NH_3_ volatilization by 18% after 14 d of incubation, while PPDA decreased NH_3_ volatilization by 9% after 10 d.

N_2_O emission from animal manure is associated with soil mineralization processes because N_2_O is generated primarily through microbial nitrification of NH_4_^+^ to NO_2_^−^ and then NO_3_^−^; and denitrification of NO_3_^−^ to N_2_O [28]. In the present study, daily N_2_O emissions ranged from 0.84 to 18.60 g N_2_O-N/ha/d. The significant reduction of N_2_O emission by DCD treatments, as estimated by 59.8% of reduction by DCD alone and 50.0% by HQ+DCD compared with the control (Figure 6B), suggested that the nitrification inhibitor DCD deactivates the enzymes responsible for the oxidation of NH_4_^+^, reducing its conversion to NO_3_^−^, which limits the pool of denitrification for N_2_O emission [5] as well as susceptible leaching [6]. The present data showed that the urease inhibitor HQ also significantly reduced N_2_O emission by 40.7% compared to the control, confirming that HQ plays an important role in reducing N_2_O emission by reducing the pool of NH_4_^+^ released from urea hydrolysis (Figure 3A), which is a primary source of nitrification and of following denitrification [5,9]. The stronger effect of nitrification inhibitors, compared with that of the urease inhibitor, on reducing N_2_O emission has also been shown in several crop fields applied with urea [9]. Nitrification inhibitors have been shown to successfully reduce N_2_O emission from various cropping systems [24,27] and pastures [26].

In this study, positive effects of HQ and/or DCD in reducing NO_3_^−^ leaching from the soil were observed, as demonstrated by 7.0%, 12.9%, and 11.8% reductions in NO_3_^−^ leaching in the soil in the HQ, DCD, and HQ+DCD plots, respectively. This result may reflect the priming effect of the inhibitors on delaying nitrification, as shown by the lower level of soil NO_3_^−^ (Figure 3C) and slightly higher NH_4_^+^ (Figure 3A). Other studies have shown that nitrification inhibitors efficiently reduced NO_3_^−^ leaching from the soil amended with NH_4_^+^-based N fertilizer (including urea-based or other organic amendments, which subsequently convert to NH_4_^+^) by retaining N in the soil NH_4_^+^ form over a longer period, reducing the peak concentration of soil NO_3_^−^ and the potential for N losses through denitrification or NO_3_^−^ leaching from the soil [30]. In addition, Zaman and Blennerhassett [14] revealed that the addition of urease inhibitor NBPT reduces NO_3_^−^ leaching to a greater extent for synthetic fertilizer and animal excreta.

In conclusion, with progressing regrowth of perennial ryegrass pasture, the uptake of applied pig slurry-urea ^15^N by herbage gradually increases, whereas soil urea ^15^N recovery decreased. The herbage urea ^15^N recovery was not affected by the application of HQ and/or DCD during the first 14 d of regrowth. However, at the final regrowth (56 d), application of HQ and/or DCD resulted in an increase in urea ^15^N recovery in both the herbage and soil, with the strongest effect observed for HQ+DCD. The conversion of pig slurry urea-derived N into soil NH_4_^+^ and NO_3_^−^ fractions were reduced by the inhibitors, with a higher effect observed for HQ during the first 14 d. The conversion of pig slurry-urea N into soil NH_4_^+^ and NO_3_^−^ fractions was enhanced especially in the presence of DCD during the latter regrowth period. Higher retention of soil inorganic N derived from pig slurry-urea at the final regrowth (56 d) in the HQ and/or DCD plots was in line with the enhanced herbage N recovery as well as the reduced N losses. The application of HQ and/or DCD resulted in the efficient reduction of NH_3_, N_2_O emission, and NO_3_^−^ leaching. Application of HQ or DCD alone also significantly reduced N losses. Therefore, it can be concluded that HQ and DCD efficiently improve the N use efficiency of pig slurry-urea, contributing a positive role in reducing N losses to the environment.

## IMPLICATIONS

Management strategies of animal manure are necessary to improve nitrogen use efficiency while minimizing N losses to environmental pollution. The application of urease inhibitor (hydroquinone) and/or nitrification inhibitor (dicyandiamide) may enhance the nitrogen use efficiency of pig slurry by delaying the hydrolysis of urea and nitrification, thereby alleviating the nitrogen losses to nitrate leaching, ammonia, and nitrous oxide emission. Appropriate utilization of urease and nitrification inhibitors for pig slurry application to the grassland would be an efficient way to improve the nitrogen use efficiency, leading to a significant reduction of nitrate leaching and hazardous gases emission to the atmosphere.

## Figures and Tables

**Table 1:** Soil chemical properties of experimental site

Items	pHwater (1:5)	EC (Ds/m)	OM (%)	Total N (%)	Available P (mg/kg)	Exchangeable cation (cmol^+^/kg)		

						K	Ca	Mg
Soil	6.2	0.69	2.21	0.14	243.3	0.21	2.67	1.74

EC, electric conductivity; OM, organic matter.

**Table 2:** Recovery percentage of pig slurry-urea 15N in herbage and soil, and calculated N loss as affected by pig slurry with urease inhibitor (HQ) and/or nitrification inhibitor (DCD) during the regrowth of perennial ryegrass sward

Date	Treatment	Herbage	Soil			N loss

			Total N	(NH_4_^+^-N)	(NO_3_^−^-N)	
Day 7	Control	4.0±0.1^a^	64.6±2.2^a^	(27.4±0.8^a^)	(4.4±0.3^a^)	31.4±2.1^a^
	HQ	4.0±0.1^a^	67.8±2.4^a^	(20.6±0.6^b^)	(2.8±0.1^b^)	28.2±2.4^a^
	DCD	3.9±0.2^a^	66.4±1.8^a^	(28.1±0.9^a^)	(3.1±0.2^b^)	29.7±1.8^a^
	HQ+DCD	3.8±0.3^a^	70.4±1.2^a^	(18.6±0.5^b^)	(2.4±0.1^c^)	25.8±1.3^a^
Day 14	Control	7.1±1.0^a^	57.6±2.2^c^	(13.2±0.5^a^)	(12.5±1.5^a^)	35.3±1.3^a^
	HQ	6.7±0.5^a^	60.6±2.3^b^	(8.0±0.5^c^)	(10.6±0.9^ab^)	32.7±2.1^a^
	DCD	7.2±0.3^a^	63.4±2.1^ab^	(10.4±0.4^b^)	(12.3±1.4^a^)	29.3±1.9^ab^
	HQ+DCD	6.1±0.2^a^	67.4±2.3^a^	(7.2±0.4^c^)	(9.3±0.7^bc^)	26.5±2.1^b^
Day 56	Control	21.2±0.7^b^	40.8±2.0^c^	(0.9±0.1^d^)	(21.5±1.4^d^)	38.0±1.9^a^
	HQ	28.2±1.7^a^	44.9±1.0^ab^	(1.5±0.1^c^)	(32.1±1.6^b^)	27.0±2.2^bc^
	DCD	26.4±1.5^a^	45.2±1.8^ab^	(2.1±0.1^b^)	(29.4±1.5^bc^)	28.4±1.4^b^
	HQ+DCD	30.2±0.9^a^	48.3±2.1^a^	(2.4±0.1^a^)	(36.2±1.7^a^)	21.6±2.2^c^

Percentage of pig slurry-urea ^15^N in the soil inorganic N (NH_4_^+^-N or NO_3_^−^-N) pool.

The values are means±standard error of four replicates.

HQ, hydroquinone; DCD, dicyandiamide.

a–d Different letters in vertical column indicate significant differences at p<0.05 according to the Duncan’s multiple range test.
